# Pro-tumor γδ T Cells in Human Cancer: Polarization, Mechanisms of Action, and Implications for Therapy

**DOI:** 10.3389/fimmu.2020.02186

**Published:** 2020-09-16

**Authors:** Ghita Chabab, Clément Barjon, Nathalie Bonnefoy, Virginie Lafont

**Affiliations:** IRCM, Institut de Recherche en Cancérologie de Montpellier, INSERM U1194, Université de Montpellier, Institut Régional du Cancer de Montpellier, Montpellier, France

**Keywords:** γδ T cells, cancer, pro-tumor functions, immunosuppression, therapy

## Abstract

The tumor immune microenvironment contributes to tumor initiation, progression and response to therapy. Among the immune cell subsets that play a role in the tumor microenvironment, innate-like T cells that express T cell receptors composed of γ and δ chains (γδ T cells) are of particular interest. Indeed, γδ T cells contribute to the immune response against many cancers, notably through their powerful effector functions that lead to the elimination of tumor cells and the recruitment of other immune cells. However, their presence in the tumor microenvironment has been associated with poor prognosis in various solid cancers (breast, colon and pancreatic cancer), suggesting that γδ T cells also display pro-tumor activities. In this review, we outline the current evidences of γδ T cell pro-tumor functions in human cancer. We also discuss the factors that favor γδ T cell polarization toward a pro-tumoral phenotype, the characteristics and functions of such cells, and the impact of pro-tumor subsets on γδ T cell-based therapies.

## Introduction

Within a tumor, the malignant features of cancer cells are tightly regulated by their local environment and the reciprocal network they form with host cells (e.g., immune cells, angiogenic vascular cells, endothelial cells, and cancer-associated fibroblasts) and that define the cancer ecosystem. The tumor immune microenvironment is a critical determinant of cancer evolution and outcome. In this context, the nature and frequency of tumor-infiltrating immune cells are considered to be prognostic factors in many cancers. A better knowledge of this dynamic immune environment is required to improve prognosis, choose therapies, and evaluate the response to treatments.

Among the tumor-infiltrating immune cells, T cell sub-populations, especially CD8+ T lymphocytes, are a key anti-tumor immune component. γδ T cells, a subgroup of T cells that belong to the non-conventional or innate lymphocyte family, also are found in the tumor microenvironment and are involved in tumor surveillance. Although they share many properties with αβ T cells, such as cytotoxic activity and pro-inflammatory cytokine production, the structure of their T cell receptor (TCR; composed of γ and δ chains) is different as well as their activation mechanisms that are independent of major histocompatibility complex (MHC) molecules. Human γδ T cells can be divided in three main populations, based on their TCR δ chain (δ1, δ2, δ3) (1, 2). Vδ2 T cells, also known as Vγ9Vδ2 T cells, are the main γδ T subtype (90%) in peripheral blood. The Vδ1 and Vδ3 subsets are mostly found in tissues and mucosa, respectively.

Vγ9Vδ2 T cells display specific properties, such as the TCR-dependent recognition of non-peptidic phosphorylated antigens, called phosphoantigens. Phosphoantigens are molecules produced by the isoprenoid synthesis pathways of prokaryotic pathogens and by infected or transformed eukaryotic cells. Although phosphoantigen recognition does not require MHC molecule presentation, several studies brought evidences of the involvement of the cell surface butyrophilin 3A (BTN3A) (3) and the requirement of butyrophilin 2A1 (BTN2A1) (4). Phosphoantigen-induced TCR activation of Vγ9Vδ2 T cells triggers their proliferation, cytokine production, and cytotoxic activity (5). Vγ9Vδ2 T cells also express natural killer (NK) receptors, such as NKG2A and NKG2D, and their activation is modulated by the presence of their ligands in the environment (6, 7).

Vδ1 T cells recognize the stress-inducible MHC class I-related chain A and B (MICA and MICB) proteins that are expressed by some tumor and virus-infected cells (8), as well as glycolipid antigens presented by the CD1c (9) and CD1d proteins (10, 11), and the algal protein phycoerythrin (12). Additionally, Vδ1 T cells can be activated independently of their TCR, via ligation of stimulatory receptors, including NKG2C, NKG2D, NKp30, toll-like receptors, and the β-glucan receptor dectin 1 (13–17). To date, little is known on the activation mechanisms of the Vδ3 T cell subset.

Although the human Vδ1, Vδ2 and Vδ3 T cell subsets display a strong reactivity against tumor cells, γδ T cell-based immunotherapies primarily target the Vδ2 subset because they are easily expanded and activated by synthetic clinical-grade phosphoantigens (e.g., bromohydrin pyrophosphate) or by pharmacological inhibitors (e.g., zoledronate) of the isoprenoid synthesis pathway that produces these metabolites (18, 19).

Many clinical trials using Vγ9Vδ2 T cells have been carried out. Although their safety have been proven, response rate was moderate and only in 10–33% of patients with hematologic and solid malignancies benefit from Vγ9Vδ2 T cell-based immunotherapies (20–25). This suggests the presence in the tumor microenvironment (TME) of suppressive mechanisms that inhibit/divert Vγ9Vδ2 T cell functions and/or their ability to infiltrate tumors. New tools to target and boost Vγ9Vδ2 T cell anti-tumor functions are currently under study (26), while other γδ T cell subtypes (e.g., Vδ1 T cells) are now tested as new therapeutic candidates (27). Although therapies using γδ T cells received a new burst of interest due to these new research axes, the existence of γδ T cell subsets with pro-tumor functions has also been suggested.

In this review, we will discuss the evidences concerning γδ T cell pro-tumor functions in human cancer, and the factors that could favor γδ T cell polarization toward a pro-tumoral phenotype, the characteristics and functions of these cells, and also the possible consequences for γδ T cell-based therapies.

## Evidence of Pro-tumoral γδ T Cells in Human Cancer (Table 1)

In line with the potent anti-tumor properties of γδ T cells, a large study of publicly available gene expression data from bulk tumors showed that the γδ T cell signature is associated with the most significant favorable prognosis in 25 malignancies (37). However, it was later demonstrated that the sorting algorithm used in this study could not accurately differentiate γδ T cells from CD8+ and NK cells due to the transcriptome overlaps in these three cell types (38). Using a refined signature for the Vγ9Vδ2 T cells subset based on sorted cells, the authors found that a high-level infiltration of γδ T cells in tumors was not always associated with a positive outcome (38). In line with these results, recent studies suggested that these cells may also have a pro-tumor role in some cancers.

**Table 1:** Pro-tumoral characteristics of infiltrating γδ T cells in human cancer.

**Type of cancer**	**γδ sub-populations**	**Phenotype (surface markers)**	**Mode of action**	**Pro-tumoral/suppression factors**	**Prognosis value**	**References**
Breast cancer	Vδ1 (predominantly)	CD8αα+, CD25–, FoxP3– (TILs clones)	Suppression of T cells and DC	Undefined soluble factor (not TGF-β or IL-10)	Correlation with advanced tumor stages, inverse correlation with OS and RFS	(28, 29)
	Vδ1 and Vδ2	CD39+, CD73+	n/a	n/a	Associated with late stage disease	(30)
Colorectal cancer	Vδ1 (predominantly)	CD39+, CD25+, FoxP3+	Suppression of T cells	Adenosine	Correlation with malignant clinicopathological features	(31)
	Vδ1 (Vδ2 defined as anti-tumoral)	n/a	Suppression of T cells	n/a	Correlation of Vδ1 with disease T stage (negative correlation with Vδ2)	(32)
	Vδ1 (predominantly)	CD45RO+, CD161+, CCR6+, CD69+ TEM phenotype CD45RA–, CD27–	Attraction of PMN-MDSCs	IL-17A, IL-8, GM-CSF	Correlation with advanced clinicopathological features	(33)
Gallbladder cancer	γδ	n/a (CXCR3)	Angiogenesis, suspected attraction of MDSCs	IL-17A	Associated with poor survival	(34)
Ovarian cancer	Vδ1 (predominantly)	n/a	Suppression of T cells, suspected promotion of pro-tumoral myeloid cells	Suppressive factor not determined, production of IL-17A	Correlation with advanced clinicopathological features	(35)
Pancreatic ductal adeno carcinoma	Non Vγ9	TEM phenotype CD45RA–, CD27–, CD62L–	Suppression of T cells (mouse model)	PD-L1, Galectin-9	n/a	(36)

In breast cancer, high Vδ1 T cell prevalence has been associated with immunosuppressive functions, such as inhibition of naive T cell proliferation and the impairment of dendritic cell (DC) maturation and function (28). Moreover, γδ T cell infiltration level in breast cancer was the most significant independent prognostic factor of disease severity, in terms of survival and relapse (29).

In colorectal cancer, CD39+ Vδ1 T cell infiltration establishes an immunosuppressive microenvironment through the adenosine pathway and the recruitment of myeloid-derived suppressive cells (MDSCs). The presence of these cells has been associated with the disease severity (31). Another study demonstrated the pro-tumor functions of IL-17-producing γδ T cells in colon cancer through their capacity to recruit MDSCs (33). Moreover, pro-inflammatory Vδ2 T cells might participate in colorectal cancer pathogenesis by supporting chronic inflammation (39). Besides breast and colon cancer, several studies have shown a potentially deleterious role of γδ T cell subsets in pancreatic, ovarian, gallbladder and renal cancer (32, 34–36).

## Polarization of γδ T Cells Toward a Pro-tumor Functional Phenotype (Figure 1)

Although γδ T cells have been originally described as pro-inflammatory cells with a Th1-like phenotype, they display high plasticity and can be polarized toward different functional phenotypes, depending on their environment (40). Understanding precisely the influence of different environmental factors, such as cytokines, on γδ T cells and the limits of their plasticity is crucial to determine how the TME can skew γδ T cells toward a pro-tumor function that will directly or indirectly impair the anti-tumor immune response and support tumor growth. Although studying T cell functional plasticity within tumors is a complex endeavor, several *ex vivo* studies involving the activation of naive γδ T cells in the presence of various cytokines have brought some insights into how γδ T cells can be skewed toward a pro-tumoral activity. Specifically, it has been shown that TGF-β, IL-4 and more recently IL-21 favor the acquisition of pro-tumoral properties by human and mouse γδ T cells. Moreover, various cytokine combinations can polarize γδ T cells into Th17-like cells with pro-tumor effects.

**Figure 1:**
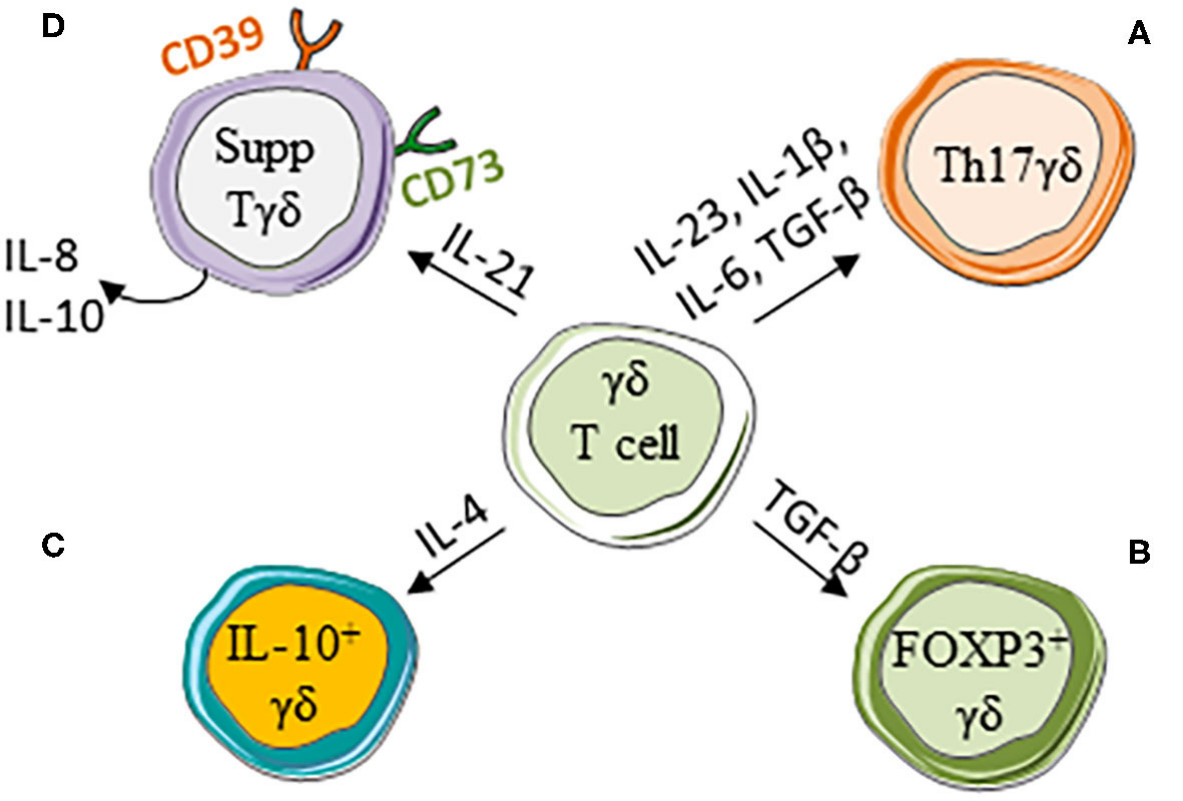
γδ T cell polarization into pro-tumor cells. Cytokines present in the tumor microenvironment induce the differentiation of γδ T cells into pro-tumor cells: **(A)** Th17-like γδ T cells (Th17 γδ), **(B)** FOXP3+γδ T cells (FOXP3+γδ), **(C)** IL-10-producing γδ T cells (IL-10+ γδ), and **(D)** regulatory γδ T cells that express CD39 and/or CD73 (Supp γδ).

### TGF-β

TGF-β is a pleiotropic cytokine that is produced by most cells in a latent form. TGF-β1 (subsequently referred to as TGF-β), the most studied isoform, is a potent suppressor of the immune system. It can be secreted in a complex with latent TGF-beta binding proteins (LTBP) and deposited in the extracellular matrix, or tethered to the surface of cells when bound in a covalent manner to glycoprotein A repetitions predominant (GARP) or leucine-rich-repeat-containing protein 33 (LRRC33). Active TGF-β needs to be released from the latent complex through the interaction with other partners, such as integrins, to act on its target cells through binding to TGF-β receptors (41, 42). TGF-β can induce the differentiation of naive CD4+ T cells into regulatory T cells (Tregs) or Th17 cells, depending on the context, and is often enriched in tumors. Therefore, TGF-β could play a crucial role in γδ T cell polarization toward pro-tumoral cells in the TME (43, 44). *In vitro*, human peripheral blood mononuclear cells (PBMCs) can be stimulated with phosphoantigens and cultured with IL-2 to selectively expand Vγ9Vδ2 T cells. Addition of TGF-β to the culture increases FOXP3 expression in these cells. FOXP3 expression remains stable for at least 10 days. Sorted FOXP3+ Vγ9Vδ2 T cells inhibit the proliferation of TCR-stimulated PBMCs (45). Another study confirmed TGF-β role in the development of FOXP3+ Vγ9Vδ2 T cells and demonstrated that decitabin, a DNA hypomethylating agent, promotes the generation and the immunosuppressive activity of FOXP3+ Vγ9Vδ2 T cells induced by TGF-β (46). Importantly, the relevance of FOXP3 as a regulatory marker depends on the type of stimulation. Indeed, Vδ2 cell activation using anti-CD3 and anti-CD28 antibodies instead of phosphoantigens leads to transient FOXP3 expression that does not correlate with the regulatory phenotype (47, 48). Interestingly, vitamin C increases the stability of TGF-β-induced FOXP3 expression in Vδ2 cells through an epigenetic modification of the FOXP3 gene, and enhances their suppressive capacities (49). Li et al. demonstrated that upon TCR stimulation Vδ1 T cells can be polarized toward a suppressive phenotype in the presence of IL-2 and TGF-β. These Vδ1 cells express FOXP3 and suppress the proliferation of activated CD4+ T cells (50). In human colorectal cancer, tumor-infiltrating CD39+ γδ T cells were described as regulatory γδ T cells that express FOXP3 and act mainly through the adenosine pathway (31). The authors found that TGF-B1 mRNA level is higher in the tumor than in the associated normal tissue. Moreover, CD39+ γδ T cells from normal tissue incubated with tumor supernatant acquire a potent suppressive capacity through increased adenosine production. This effect can be abrogated by incubation with an anti-TGF-β antibody, and can be reproduced by stimulating cells with recombinant TGF-β. TGF-β-induced polarization of γδ T cells toward FOXP3+ suppressive cells was also demonstrated in the mouse (51). Additionally, TGF-β is required for the polarization of Vγ9Vδ2 into IL-17-producing γδ T cells, together with IL-1β, IL-6 and IL-23, as described below (52). Overall, these results suggest that TGF-β could be one of the key factors responsible for conversion of γδ T cells into suppressive and/or IL-17-producing cells.

### IL-4

IL-4 is a potent regulator of the humoral response and more generally of the adaptive immunity, particularly through the differentiation of naive T cells into Th2 cells. In cancer, IL-4 has been associated with tumor aggressiveness, and IL-4 pathway blockade is currently investigated as anti-cancer strategy (53). IL-4 is often enriched in the microenvironment of human solid tumors, notably in cancers with high γδ T cell infiltration, such as breast cancer (54). *In vitro*, human Vδ2 cells isolated from peripheral blood and activated by phosphoantigens in the presence of IL-4 produce low levels of interferon γ (IFN-γ) and high levels of IL-4, although this production is not stable over time (55). In a more recent study, Mao et al. showed that IL-4 inhibits *in vitro* the activation of blood γδ T cells induced by TCR stimulation (54). Nevertheless, IL-4 promotes the growth of activated γδ T cells and increases the levels of Vδ1 T cells, which in turn inhibit Vδ2 T-cell growth via significant IL-10 secretion (54). IL-4 inhibits γδ T cell activation when present at the moment of the stimulation, but enhances their proliferation when added later. Moreover, concanavalin A-stimulated Vδ1 T cells cultured with IL-4 retain their cytotoxic properties against tumor cells. This suggests a complex and context-dependent role of IL-4 in γδ T cell polarization (56).

### IL-21

IL-21 is a potent immunomodulatory cytokine, mainly produced by activated CD4+ T cells and NKT cells. IL-21 enhances the effector functions of NK cells, helper CD4+ T cells and cytotoxic T cells (CTL), but also inhibits Tregs (57). Therefore, it is often defined as a pro-inflammatory cytokine. In colorectal cancer, IL-21 is strongly associated with chronic inflammatory colitis that precedes the malignant disease (57–59). A similar pro-inflammatory effect of IL-21 on γδ T cells was initially described. Upon *in vitro* expansion with IL-21, human Vγ9Vδ2 cells display increased levels of granzyme B and increased production of IFN-γ after activation, resulting in enhanced cytotoxic activity toward tumor cells (60). However, IL-21 modulatory role may depend on the cell type and the duration of the exposure. For example, IL-21 enhances IL-10 production by regulatory B cells and their proliferation. Similarly, our group recently found that IL-21 is implicated in the polarization of human Vγ9Vδ2 T cells and Vδ1 T cells toward a regulatory phenotype (30, 61). We isolated a subpopulation of CD73+ regulatory Vγ9Vδ2 T cells following their expansion in the presence of IL-21. We demonstrated that this subset can synthetize adenosine through CD73 enzymatic activity, and produces the suppressive cytokine IL-10 and the chemokine IL-8 (also known as CXCL8) that is involved in the recruitment of polymorphonuclear leukocytes (PMN)-MDSCs. This CD73+ cell subpopulation can suppress the T cell immune response directly in an adenosine- and IL-10-dependent manner, and indirectly by impairing DC antigen presentation (61). We then extended these observations to Vδ1 T cells. We identified in the blood of healthy donors a Vδ1 T cell subpopulation that expresses CD73 and displays immunosuppressive phenotype and functions (i.e., production of immunosuppressive molecules, such as IL-10, adenosine and IL-8). As shown for Vγ9Vδ2 T cells, incubation with IL-21 favors the development and amplification of this Vδ1 subset. Importantly, we detected CD73+ γδ T cells in breast cancer biopsies, suggesting that they could interfere with the anti-tumor response (30). Moreover, in mouse γδ T cells, CD73 expression is increased after exposure to IL-21, suggesting that this polarization could be a common mechanism among different species (61). Interestingly, after infection with Mycobacterium bovis Bacillus Calmette-Guerin (BCG), the number of IL-17-producing γδ T cells was higher in IL-21 receptor knockout mice than wild type animals. IL-21 induces the apoptosis of these cells, suggesting the existence of a balance between IL-21-induced regulatory γδ T cells and IL-17-producing γδ T cells, at least in some contexts (62).

### Polarization Into Th17-Like Cells

IL-17 production was first described in helper CD4+ cells, called Th17 cells. Th17 cell cytokine secretion, transcription regulation and effects on the immune system are now well-characterized. Their development is controlled by the transcription factors RORγt (63) and STAT3, and also by IRF4 in some cases when the differentiation is induced by cytokines (64). In mice, TGF-β, IL-6, IL-21 and IL-23 play a critical role in the differentiation or polarization of CD4+ cells into Th17 cells. In humans, IL-1 and IL-23 seem to have the most important role in Th17 cell differentiation, followed by TGF-β and IL-6 (65–67). IL-17 is produced by murine γδ T cells (68) and also by human γδ T cells (69). In both species, IL-7 strongly promotes the expansion of IL-17-producing γδ T cells (Th17 γδ T cells) (70). Moreover, several studies have shown that when cultured in the presence of various cytokine combinations, naive Vγ9Vδ2 T cells acquire an IL-17-secreting Th17-like phenotype or a mixed Th1/Th17 phenotype, and produce both IFN-γ and IL-17 (52, 71, 72). Human cord blood-derived Vγ9Vδ2 T cells stimulated with the phosphoantigen (E)-4-hydroxy-3-methyl-but-2-enyl pyrophosphate (HMBPP) require IL-6, IL-1β and TGF-β to differentiate into Th17 γδ cells, and also IL-23 for differentiation into γδ Th1/Th17 cells (71, 72). In adults, differentiation of naive γδ T cells into memory γδ Th1/Th17 T cells and Th17 γδ T cells requires IL-23, IL-1β and TGF-β, but not IL-6. γδ Th17 cells can also produce IL-22 (especially cells in the cord blood) (71, 72). The pro-tumor role of IL-17 has been well established in some contexts, and the pro-tumor role of Th17 γδ T cells will be developed in the next part.

## Pro-tumoral Functions of γδ T Cells (Figure 2)

**Figure 2:**
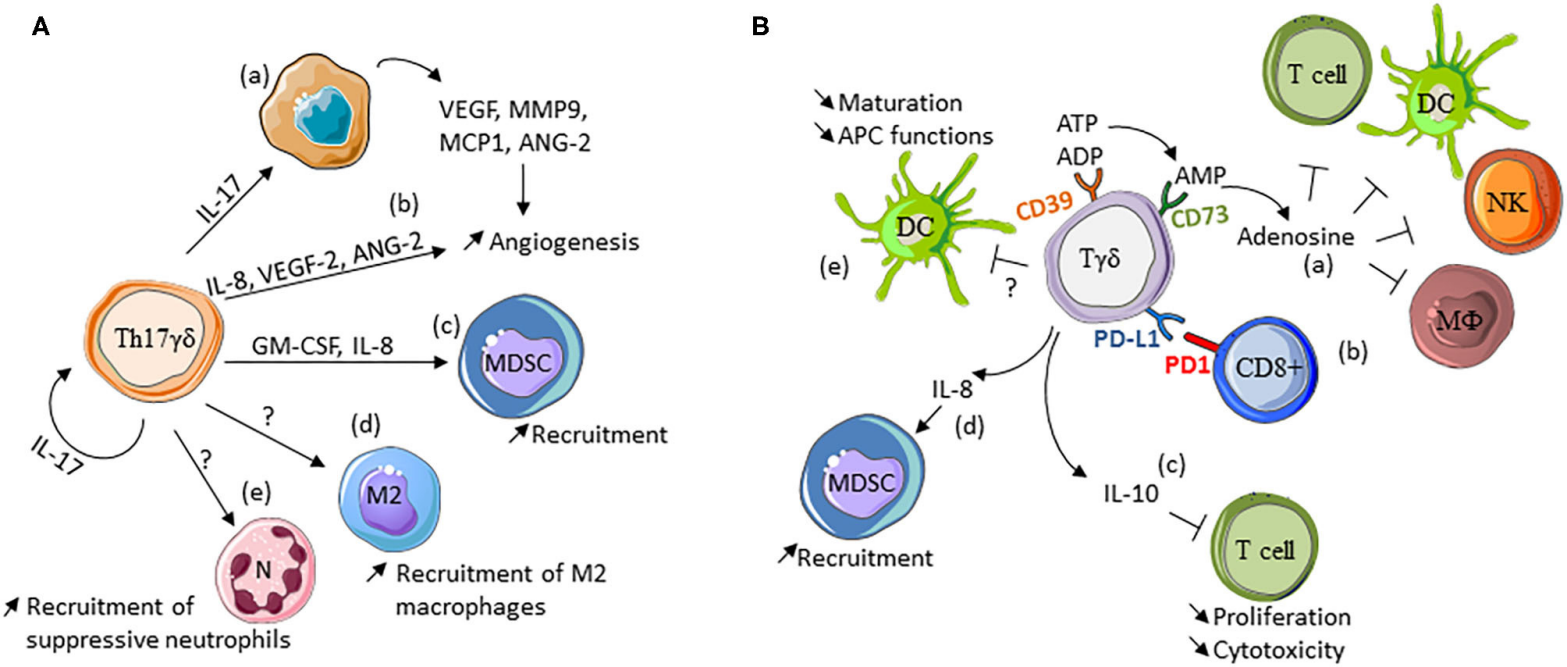
Pro-tumor functions of γδ T cells. **(A)** Th17-like γδ T cells. Th17γδ cells promote angiogenesis indirectly by inducing the production of angiogenic factors by cancer cells (a), or directly by producing angiogenic factors (b). They also induce myeloid-derived suppressor cell (MDSC) recruitment through GM-CSF and IL-8 production (c), and recruit or polarize M2 macrophages (d) and suppressive neutrophils (e). **(B)** Pro-tumor γδ T cells. CD39+/CD73+ γδ T cells produce adenosine that inhibits αβ T cell proliferation and anti-tumor functions (a). γδ T cells can express PD-L1 and inhibit the function of PD1-expressing cells, such as CD8 + cells (b). IL-10 produced by γδ T cells inhibits CD8 + cell proliferation and cytotoxicity (c). They also favor MDSC recruitment via IL-8 (d) and inhibit dendritic cell (DC) maturation and functions through not identified mechanisms (e). NK, natural killer cells; Mϕ, macrophages, APC, antigen-presenting cell.

### Th17 γδ T Cells

IL-17 is detected in mice and human tumor (73–75), and αβ Th17 cells are not the only source of IL-17. Indeed, NK cells, neutrophils and γδ T cells also produce IL-17. Notably, Th17 γδ T cells are the first and major source of IL-17 at sites of inflammation or infection, and also in tumors.

Although Ma et al. showed that IL-17-producing γδ T cells (Vγ4 and Vγ6) contribute to the chemotherapy-induced anti-cancer immune response (76), many studies found that Th17 γδ cells display pro-tumor functions in mouse models and human solid cancers.

In mouse models of fibrosarcoma (77), ovarian (78) and breast cancers (79), γδ T cells are the main IL-17 producers at the tumor site, and promote tumor growth. Th17 γδ T cells increase the expression of the angiogenic factors VEGF-2 and ANG-2 at the tumor sites, suggesting that tumor-infiltrating IL-17-producing γδ T cells promote tumor development by enhancing angiogenesis (77). They also participate in the establishment of an immunosuppressive TME through the recruitment, expansion and polarization of neutrophils that can suppress cytotoxic T lymphocyte (CTL) activities (79), and the recruitment of MDSCs or small peritoneal macrophages in ovarian cancer. All these cells also induce the expression of pro-tumor and pro-angiogenic factors that promote tumor growth.

In human solid cancers, Wu et al. were the first to demonstrate the pro-tumor role of IL-17-producing γδ T cells in human colorectal cancer (33). They showed that the main IL-17 producers in colon cancer are γδ T cells (up to 83% of Vδ1 T cells). In this cancer, Th17 γδ T cell differentiation and activation are triggered by IL-23 produced by activated DCs present at the tumor site. Colon cancer-infiltrating Th17 γδ T cells produce also IL-8 that participates in tumor progression through its role in angiogenesis and in MDSC recruitment. These MDSCs contribute to establishing an immunosuppressive microenvironment that favors tumor development. Interestingly, the strong and positive correlation between tumor-infiltrating Th17 γδ T cells and TNM stage (tumor size, lymphatic invasion, and metastases) strengthens the pro-tumor activities of Th17 γδ T cells in human colorectal cancer (33). Studies in patients with gallbladder cancer showed an increase of Th17 γδ T cells in the blood (compared with healthy individuals), and also of tumor-infiltrating lymphocytes in patients who did not receive any treatment. They confirmed the implication of Th17 γδ T cells in angiogenesis promotion (induction of VEGF production by gallbladder cancer cells) and tumor progression. Moreover, the presence of Th17 γδ T cells in the blood of patients is associated with poor survival compared with patients with few or without Th17 γδ T cells (34). Lo Presti et al. showed that γδ T cells are increased in the blood and at the tumor site in patients with squamous cell carcinoma. Interestingly, tumor-infiltrating γδ T cells are functionally different depending on the tumor stage (80). At early stages, γδ T cells produce mainly IFN-γ, while at late stages, they produce IL-17. Indeed, higher numbers of IL-17-producing cells (both Vδ1 and Vδ2 γδ T cell subsets) are found in advanced-stage squamous cell carcinoma compared with early stage tumors. They also showed that both Vδ1 and Vδ2 cell subsets produce high levels of IL-17 at the tumor site. Moreover, Vδ2 T cells produce IFN-γ in the blood, suggesting that Th17 γδ T polarizing factors are present in the TME (80).

Overall, many reports demonstrated the pro-tumor functions of γδ T cells with a Th17 γδ T phenotype. To date, it is not possible to say whether this Th17 γδ T cell sub-population is recruited at the tumor site or is polarized *in situ* toward IL-17-producing cells due to the presence of Th17-polarizing cytokines in the TME (e.g., IL-1β, IL-23, TGF- β, IL-6). Nevertheless, it is now well-established that Th17 γδ T cells favor tumor growth by promoting angiogenesis, metastasis development, and the recruitment of other immunosuppressive cells, such as suppressive neutrophils and MDSCs.

### Production of Suppressive Cytokines

As discussed in the polarization section, upon exposure to specific stimuli γδ T cells can acquire potent regulatory functions, particularly through the production of IL-10 and TGF-β, two strongly suppressive cytokines.

IL-10 is a key anti-inflammatory cytokine that inhibits the production of pro-inflammatory cytokines and the expression of co-stimulatory molecules by Th1 and antigen-presenting cells (81). *In vitro*, IL-4-polarized Vδ1 T cells produce IL-10 and inhibit the growth of Vδ2 T cells in an IL-10-dependent manner. Similarly, Vδ1 T cells activated with anti-TCR antibodies strongly secrete IL-10 (54, 82). In the presence of IL-21, the CD73+ Vδ2 and Vδ1 T cell subsets secrete high levels of IL-10 upon activation (30, 61). In human colorectal cancer, infiltrating CD39+ γδ T cells, which are mainly Vδ1+ cells, produce more IL-10 than CD39- γδ T cells and CD39+ γδ T cells from the tumor-adjacent normal tissue. However, after several days of culture *ex vivo*, these cells do not maintain IL-10 production and lose their ability to suppress the proliferation of activated T cells (31). In mice, IL-10-producing γδ T cells have been identified in tumors. In a breast cancer model, supernatant from infiltrating γδ T cells suppresses the proliferation of anti-tumor CTLs in an IL-10-dependent manner (83). In a syngeneic model of OVA-expressing EL4 tumors (lymphoma), IL-10-producing γδ T cells suppress the CD8-dependent anti-tumor response, and their depletion significantly reduces tumor growth (84). Similarly, IL-10+ γδ T cells are observed in the spleen and tumors of mice grafted with TC1 cells (transformed lung epithelial cells) (61). IL-10-producing γδ T cells are also observed in other conditions, for instance during pregnancy (both human and mouse), and in oral tolerance and infection in the mouse (85–87). Collectively, these results suggest that Vδ1 and Vδ2 T cells can produce IL-10; however, the amount and the impact of this production in human tumors has not been clearly established yet.

TGF-β is a potent immunosuppressive factor that is tightly regulated, particularly at the post-translational level. To be active, the mature part of the protein needs to be released from the latent peptide (LAP) through interaction with the integrin αvβ6 or αvβ8, the main activating partners of TGF-β. *In vitro*, TGFβ mRNA level and LAP surface expression are increased in Vδ1 T cells sorted from PBMCs and activated with anti-CD3 and anti-CD28 antibodies (88). High TGF-β level has also been detected in the supernatant of PBMCs stimulated with an anti-TCR Vδ1 antibody (82), and in the supernatant of Vδ2 T cells stimulated with the ligand isopentenyl pyrophosphate and expanded with TGF-β and IL-15 (45). In colorectal cancer, TGF-β surface expression is higher in γδ T cells isolated from tumors than from normal tissue (31). Interestingly, in the mouse tumor model MM2, infiltrating γδ T cells suppress the anti-MM2 CTLs through TGF-β in addition to IL-10 (83). However, it is unclear whether total or active TGF-β was measured in these studies. While total TGF-β is a measure of the whole TGF-β production by the cells, only active TGF-β quantification indicates the actual suppressive potential of such cells through TGF-β. Indeed, in these studies, γδ T cell suppressive properties were not affected by a neutralizing anti-TGF-β antibody, despite their supposed high level of TGF-β production, or the impact of TGF-β neutralization was not explored. A possible explanation for this discrepancy is that only total TGF-β was measured and not active TGF-β. This argument is supported by the reported high concentration that is more consistent with the measurement of total TGF-β. These results suggest that human γδ T cells, particularly Vδ1 T cells, can produce and present latent TGF-β at their surface in some contexts. However, because of the lack of αvβ6 or αvβ8 integrin expression, γδ T cells might not be able to produce active TGF-β on their own, unlike conventional Tregs (89, 90). Nonetheless, the presence of latent TGF-β at the γδ T cell surface is highly relevant because they represent a new source of latent TGF- β that may be activated by integrin-expressing partners within the tumor.

Besides the production of directly suppressive cytokines, γδ T cells also support the establishment of a suppressive TME through the production of other cytokines, such as IL-8 and granulocyte macrophage-colony stimulating factor (GM-CSF) that favor PMN-MDSC accumulation and expansion in colorectal cancer (33). Interestingly, IL-21, which is highly expressed in this cancer type, increases the production of IL-8 by CD73+ Vδ2 T cells and Vδ1 T cells *in vitro* (30, 61).

### Involvement of the Adenosine Pathway

Extracellular ATP and adenosine are considered potent modulators of the anti-tumor immune response. Extracellular ATP, released by apoptotic cells for example, induces inflammation and promotes strong anti-tumor responses because it increases the immunogenicity of dying cancer cells (91, 92). It favors the recruitment of phagocytes, the recruitment and maturation of DC, inhibits the proliferation of tumor cells but not of healthy cells, and promotes cancer cell death (91, 93, 94). Conversely, extracellular adenosine inhibits the anti-tumor immune response and induces the establishment of an immunosuppressive microenvironment (95). The adenosine pathway involves the ectonucleoside triphosphate diphosphohydrolase 1 (ENTPD1 or CD39) that catalyzes the phosphohydrolysis of extracellular ATP into ADP and of ADP into AMP, and the ecto-5′-nucleotidase CD73 that completes AMP conversion into adenosine (92, 96, 97). It has been shown that γδ T cells express CD39 and/or CD73 during inflammation and in the TME. Their expression is associated with suppression or inhibition of the immune response (98–100). In murine pancreatic cancer, Daley et al. found that tumor-infiltrating γδ T cells upregulate CD39 expression (among other immunosuppressive molecules) and promote tumor progression by restricting αβ T cell activation (36). Hu and colleagues described in human colorectal cancer a subpopulation of regulatory γδ T cells that express CD39 (31). CD39+ γδ T cells are enriched at the tumor site and produce high levels of adenosine in the TME, compared with other regulatory cells such as conventional Tregs. Furthermore, they showed that infiltration of CD39+ γδ T cells is positively correlated with the TNM stage, suggesting that these cells participate in the establishment of an immunosuppressive TME, thus promoting tumor growth (31). *In vitro*, our group identified subpopulations of regulatory γδ T cells isolated from peripheral blood that express CD73 and can produce adenosine. These CD73+ populations (Vγ9Vδ2 or Vδ1) also express CD39 and catalyze the transformation of ATP into adenosine, thus displaying immunosuppressive functions, as revealed by their capacity to inhibit αβ T cell proliferation (30, 61). These regulatory CD73+ γδ T cells are found in human breast cancer samples, suggesting that they could interfere with the anti-tumor immune response and favor tumor progression (30). Altogether, these studies indicate that the CD39/CD73/adenosine pathway is a major component of γδ T cell regulatory/immunosuppressive functions in the TME.

### Other Suppressive Mechanisms of γδ T Cells

The previously described regulatory γδ T cells can contribute to the establishment of an immunosuppressive microenvironment and to the inhibition of the anti-tumor response in different manners, for instance by producing inhibitory factors (e.g., IL-10, IL-8, TGF-β and adenosine) or by recruiting immunosuppressive cells (e.g., MDSCs and neutrophils). γδ T cells can also exert their regulatory functions by providing negative co-stimulatory signals to T cells in the TME through expression of immune checkpoint proteins. Programmed cell death 1 (PD1) and its ligand programmed cell death 1 ligand 1 (PD-L1) play a major role in the negative regulation of cell-mediated immune responses. Indeed, PD1 is expressed by T cells, and upon binding to its ligand (expressed by B cells, macrophages and cancer cells), it inhibits T cell activation, thus impairing the anti-tumor T cell response. Peters et al. showed that Vδ2 T cells obtained from the blood of healthy donors can express PD-L1 following activation (47). These cells inhibit αβ T cell proliferation in co-culture experiments, and this effect can be abrogated by PD-L1 blockade (47). This could be another mechanism by which regulatory γδ T cells exert their immunosuppressive activities and promote tumor growth. In agreement, Daley et al. showed in a pancreatic cancer mouse model that PD-L1 expression is higher in tumor-infiltrating γδ T cells than in splenic γδ T cells (36). In co-culture experiments, they found that tumor-infiltrating γδ T cells prevent αβ T cell activation and that this inhibition is reversed by an anti-PD-L1 antibody (36). Interestingly, the same regulatory phenotype is observed in pancreatic ductal adenocarcinoma (PDAC). Indeed, PD-L1 is strongly expressed in γδ T cells from the blood of patients with pancreatic cancer compared with healthy donors. Tumor-infiltrating γδ T cells also express PD-L1 in human PDAC (50% of infiltrating γδ T cells), suggesting that γδ T cells can promote tumor progression through the PD1/PD-L1 axis (36).

T-cell immunoglobulin mucin receptor 3 (TIM-3) and its ligand galectin-9 (GAL-9) are other immune checkpoint molecules that participate in T cell response inhibition. TIM-3 interaction with GAL-9 limits T cell expansion and effector function in the TME (101, 102). GAL-9 expression is upregulated on tumor-infiltrating γδ T cells in human and mouse PDAC, and γδ T cell-mediated suppression is dependent on GAL-9 (36).

Little is known about the expression of other immune checkpoint molecules, such as PD-L2, CD80/86 and CTLA-4, by γδ T cells in cancer. More studies are needed to investigate the expression of these and other suppressive molecules to fully understand the mechanisms of action of regulatory γδ T cells.

## Implications for γδ T Cell-Based Tumor Immunotherapy

The discovery of γδ T cell-mediated tumor immune surveillance has led to much research to understand the underlying mechanisms and to harness their potent anti-tumor properties. It is now firmly established that γδ T cells are well-equipped to recognize and eliminate malignant cells (20, 103). Thus, much effort has focused on the development of therapeutics using γδ T cells, especially the Vγ9Vδ2 subset because they can be easily obtained and expanded from the blood (104, 105). Two main strategies were first investigated: (i) *in vivo* expansion of Vγ9Vδ2 T cells by injection of phosphoantigens and low-dose IL-2 in the patient, and (ii) adoptive transfer of *ex vivo* expanded Vγ9Vδ2 T cells. Clinical trials using both strategies in patients with hematological or solid cancers confirmed the safety of this immunotherapy (well-tolerated and no toxicity), but showed moderate clinical success (106–109). Indeed, the results were not as good as expected because only few patients showed complete response to the therapy. Among the reasons of these relatively modest clinical results were the skewing of γδ T cells toward a non-reactive or even a pro-tumor phenotype. For example, Hoeres et al. showed that incubation of PBMCs from patients with leukemia with IL-2 and/or zoledronic acid, which are used to activate γδ T cells, induces PD-1 expression by γδ T cells and impairs their anti-tumor functions (110). Similarly, Castella et al. reported PD-1 expression by γδ T cells in patients with myeloma after phosphoantigen activation (111). Several *in vitro* and *in vivo* studies, summarized here, have demonstrated that γδ T cell polarization toward suppressive and/or IL-17-producing cells is a real possibility and that anti- and pro-tumor γδ T cells might co-exist in the tumor.

After these first clinical trials, new refined approaches based on recent discoveries are currently being developed. Aminobisphosphonate activation of γδ T cells in combination with chemotherapy or with FDA-approved antibodies is one of these axes. Hoeres et al. and Castella et al. showed that incubation with an anti-PD-1 antibody restores the proliferative and anti-tumor properties of Vγ9Vδ2 T cells from patients with leukemia or lymphoma (110, 111). However, Castella et al. then found that phosphoantigen stimulation of anergic PD-1+ Vγ9Vδ2 combined with PD-1 blockade increases the expression of PD-1 and of two other immune checkpoint molecules (TIM-3 and LAG-3), leading to a “super-anergic” state (112). Thus, although the combination of γδ T cell stimulation and immune checkpoint blockade is an interesting and easily feasible therapeutic alternative, it still needs to be improved, by combining for example two or more antibodies against immune checkpoint molecules. The use of bi-specific T-cell engagers (BITEs), tribodies, and engineered T cells harboring a chimeric antigen receptor (CAR) are other interesting options. For instance, the redirection of Vγ9Vδ2 T cells against tumor cells using bispecific antibodies or tribodies is efficient in HER-2-positive PDAC and ovarian cancer (113). TEGs are αβ T cells engineered to express tumor-specific Vγ9Vδ2 TCRs. In *in vitro* models and in humanized mouse cancer models, TEGs reduce colony formation of progenitor cells of primary acute myeloid leukemia blasts and inhibit leukemia growth (114). TEGs engineered from patients with myeloma can recognize and efficiently kill myeloma cells in a 3D bone marrow niche model. Phase 1 clinical trials are currently in development to test TEGs, CAR γδ T cells, and antibodies (bispecific antibodies or anti-BTN3A antibodies) to specifically “engage” γδ T cells in the anti-tumor immune response (26).

Another strategy would be to focus on Vδ1 T cells, the main subpopulation that infiltrates the TME of solid tumors. Despite their potent anti-tumor properties, Vδ1 T cells had never been tested in the clinic due to lack of suitable expansion/differentiation protocols. Recently, Silva-Santos' team developed a new and robust clinical-grade method for selective and large-scale expansion and differentiation of cytotoxic Vδ1 T cells, and showed that these cells can inhibit tumor growth and dissemination in preclinical models of chronic lymphocytic leukemia (27).

On the basis of reports demonstrating γδ T cell pro-tumor functions, regulatory γδ T cell subsets could be a thorn in the side of these newly developed therapies and need to be taken into account. Unfortunately, no clear phenotypic marker of such cells has emerged yet. Vδ1 cells have been associated with pro-tumor T cells, but when cultured in proper conditions they show very high potential for anti-tumor therapies due to their strong reactivity and cytotoxicity toward tumor cells. Adenosine pathway markers (e.g., CD39 and CD73) are interesting, but do not characterize pro-tumor γδ T cells on their own. Indeed, CD39 can be considered as an activation marker for T cells (115, 116), and CD73 is also expressed by naive γδ T cells (117, 118). More studies are needed to better characterize γδ T cell pro-tumor phenotypes and to identify markers or marker combinations that will allow the depletion of pro-tumor subsets in the whole γδ T cell population.

In the absence of such specific phenotypic markers to deplete or sort out the pro-tumor γδ T cells before cell therapy, targeting polarizing cytokines or pro-tumor cytokines produced by pro-tumor γδ T cells could be of interest. While IL-21 expression might favor the emergence of a regulatory γδ T cell population, its positive role on the cytotoxicity of other cell types, such as CTL and NK cells, might be important for the anti-tumor response. Alternatively, targeting TGF-β as a pro-tumor cytokine and a polarizing factor for γδ T cells toward both suppressive and IL-17-producing cells might be of interest. Newly developed highly selective approaches targeting the TGF-β-anchoring protein GARP or the latent TGF-β peptide LAP could be employed in pro-tumor γδ T cell-rich tumors, such as colorectal cancer, or with γδ T cell-based therapies to avoid their polarization (119, 120). While no anti-human IL-10 antibody has been approved for cancer treatment, the production of IL-17A and adenosine could be targeted in tumors that are highly infiltrated by pro-tumor γδ T cells, such as breast and colorectal cancer.

## Concluding Remarks

Although γδ T cells offer interesting perspectives for clinical applications in cell-based immunotherapy, their pro-tumor functions have to be taken into account. Indeed, environmental factors can polarize or repolarize γδ T cells, leading to loss of the anti-tumor function. Moreover, important advances in γδ T cell immunobiology have revealed a large diversity in functionality and activation modes of these cells. The new challenge is to better characterize and understand the role of the various γδ T cell subsets in function of the specific context.

## Author Contributions

GC, CB, and VL wrote the initial draft. GC prepared the figures and CB the table. GC, CB, VL, and NB reviewed the manuscript. All authors contributed to the article and approved the submitted version.

## Conflict of Interest

The authors declare that the research was conducted in the absence of any commercial or financial relationships that could be construed as a potential conflict of interest.
